# In vitro and in vivo anthelmintic and chemical studies of *Cyperus rotundus* L. extracts 

**DOI:** 10.1186/s12906-023-03839-7

**Published:** 2023-01-19

**Authors:** Eman S. El-Wakil, Shimaa Shaker, Tarek Aboushousha, El-Sayed S. Abdel-Hameed, Ezzat E. A. Osman

**Affiliations:** 1 Department of Parasitology, Theodor Bilharz Research Institute, Kornaish El-Nile St, 12411 Giza, Egypt; 2 Department of Pathology, Theodor Bilharz Research Institute, Kornaish El-Nile St, 12411 Giza, Egypt; 3 Department of Medicinal Chemistry, Theodor Bilharz Research Institute, Kornaish El-Nile St, 12411 Giza, Egypt

**Keywords:** *Cyperus rotundus*, anti-*T. spiralis*, Albendazole, Mice, electron microscopy, LC-ESI-MS

## Abstract

**Background:**

Trichinellosis, a zoonosis caused by the genus *Trichinella*, is a widespread foodborne disease. Albendazole, one of the benzimidazole derivatives, is used for treating human trichinellosis, but with limited efficacy in killing the encysted larvae and numerous adverse effects. *Cyperus rotundus* L. is a herbal plant with a wide range of medicinal uses, including antiparasitic, and is frequently used in traditional medicine to treat various illnesses.

**Methods:**

LC-ESI-MS was used to identify the active phytoconstituents in the methanol extract (MeOH ext.) of the aerial parts of *C. rotundus* and its derivate fractions ethyl acetate (EtOAc fr.), petroleum ether (pet-ether fr.), and normal butanol (n-BuOH fr.). The in vivo therapeutic effects of *C. rotundus* fractions of the extracts were evaluated using the fraction that showed the most promising effect after detecting their in vitro anti-*Trichinella spiralis* potential.

**Results:**

*C. rotundus* extracts are rich in different phytochemicals, and the LC-ESI-MS of the 90% methanol extract identified 26 phenolic compounds classified as phenolic acids, flavonoids, and organic acids. The in vitro studies showed that *C. rotundus* extracts had a lethal effect on *T. spiralis* adults, and the LC_50_ were 156.12 µg/ml, 294.67 µg/ml, 82.09 µg/ml, and 73.16 µg/ml in 90% MeOH ext., EtOAc fr., pet-ether fr. and n-BuOH fr., respectively. The n-BuOH fr. was shown to have the most promising effects in the in vitro studies, which was confirmed by scanning electron microscopy. The in vivo effects of n-BuOH fr. alone and in combination with albendazole using a mouse model were evaluated by counting adults in the small intestine and larvae in the muscles, in addition to the histopathological changes in the small intestine and the muscles. In the treated groups, there was a significant decrease in the number of adults and larvae compared to the control group. Histopathologically, treated groups showed a remarkable improvement in the small intestine and muscle changes. Remarkably, maximal therapeutic effects were detected in the combination therapy compared to each monotherapy.

**Conclusion:**

Accordingly, *C. rotundus* extracts may have anti-*T. spiralis* potential, particularly when combined with albendazole, and they may be used as synergistic to anti-*T. spiralis* medication therapy.

**Supplementary Information:**

The online version contains supplementary material available at 10.1186/s12906-023-03839-7.

## Background

Trichinosis or trichinellosis is an emerging and re-emerging zoonotic parasitic disease with a considerable worldwide distribution [1]. Human infection is caused mainly by *Trichinella spiralis*, the first species discovered, following ingestion of raw or undercooked pork [2, 3]. *Trichinella spiralis* has two lifecycle phases. The intestinal phase occurs when adults colonize the small intestine during the first week after infection. In this phase, there is significant pathological damage to the villi, demonstrated as nausea, diarrhea, fever, and vomiting. After that, the muscular phase develops where the larvae invade the muscles and encapsulate. The patient complains of myalgia and muscle weakness during this phase [2, 4].

Benzimidazole derivatives, including albendazole (ABZ) and mebendazole, are the primary anthelminthic drugs used to treat this infection; however, they have a limited effect on encysted larvae [2]. Moreover, they have poor bioavailability and high resistance [5]. Furthermore, most of them are contraindicated in pregnancy and children under three [6]. These drawbacks highlight the necessity for new, effective, safe drugs against trichinellosis. Additionally, natural agents may be a promising option, having proven to be affordable, less toxic, and with no side effects, as observed with synthetic drugs [6].

Around the world, numerous medicinal herbs have been used to treat parasitic infections for hundreds of years [7]. *Cyperus* contains nearly 600 species belonging to the Cyperaceae family [8]. *Cyperus rotundus L.* “Nut” grasses are widely distributed in tropical and subtropical regions [9]. It is native to Asia, Africa, the USA, and southern and central Europe [10]. Furthermore, it has been used in folk medicine as a diuretic, aphrodisiac, sedative, carminative, and a remedy for renal colic and dysentery [11, 12]. Also, *C. rotundus* has many biological and pharmacological activities (in vitro and in vivo) such as cytotoxic [13], antimicrobial [14], anti-inflammatory [15], anti-allergic [16],anti-diarrheal [17], and hepatoprotective [18]. The previous phytochemical studies of *C. rotundus* have revealed the presence of several types of secondary metabolites, such as sesquiterpenes, flavonoids, iridoids, phenylpropanoids, furochromones, phenolic acids, alkaloids, steroids, and saponins [8, 19–21].

Several previous studies documented the anti-parasitic activity of *C. rotundus* in vitro or in vivo. The in vitro anthelmintic activity of *C. rotundus* against *Pheretima Posthuma* was reported by Kasala et al. [22]. In addition, *C. rotundus* revealed potent activity against *Plasmodium falciparum* [23] and *Entamoeba histolytica* trophozoites in vitro and were safe for use [24]. The in vivo anti-cryptosporidiosis and anti-toxoplasmosis effects of *C. rotundus* extract were reported by Fahmy et al. [25].

This study aimed to carry out qualitative phytochemical screening for the primary constituents of 90% methanol extract and its fractions with the estimation of their phenolic and flavonoid contents, characterization of the active phytoconstituents of the 90% methanol extracts, and evaluation of the anti-trichinellosis effects of *C. rotundus* extracts (in vitro and in vivo).

## Methods

### Plant materials

**Fig. 1:**
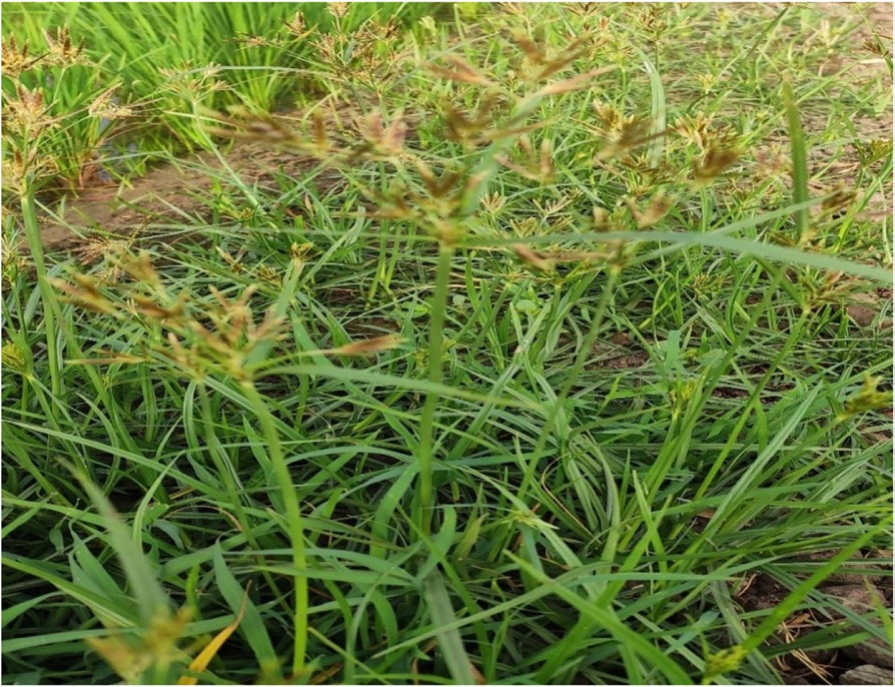
A photograph of *Cyprus rotundus*

*Cyperus rotundus* aerial parts were collected from El-Sharkia Governorate, Egypt, in June 2020. The plant sample was characterized by Mrs. Teraza Labib, a taxonomy specialist at Orman Herbarium Garden, and Prof. Mohamed El-Gebaly, Professor of Plant Taxonomy and Botany at the National Research Center, Giza, Egypt, who confirmed the identification of the plant sample. The voucher specimen (No. 20200724) of the plant (Fig. 1) was kept at the Medicinal Chemistry Department of the Theodor Bilharz Research Institute (TBRI). The plant materials were cut into small pieces, dried in the shed, and then powdered using a plant grinding machine.

### Extraction and fractionation process

*Cyperus rotundus* aerial parts dry powder (350 g) was extracted using 90% MeOH (5 × 3 L) in a 5 L conical flask. Then it was carefully closed and kept for 72 h. The supernatant was filtered using Whatman filter paper No.1 and evaporated using a rotary evaporator (Buchi, Switzerland) under a vacuum to obtain the crude extract (50 g). The dried 90% MeOH extract (40 g) was dissolved in 100 ml of water and successively partitioned with pet-ether (7 × 500 ml), EtOAc (7 × 500 ml), and n-BuOH (7 × 500 ml). The solvents were evaporated using a rotary evaporator under pressure to yield a pet-ether fraction (5.3 g), an EtOAc fraction (4.7 g), an n-BuOH fraction (7.2 g), and a residue (20.5 g). The 90% MeOH extract, and its derived fractions were kept in brown vials for further biological and chemical experiments.

### Phytochemical screening

The qualitative estimation of the major chemical compositions of *C. rotundus* extracts, such as carbohydrates, flavonoids, alkaloids, terpenoids, phenols, tannins, saponins, and glycosides, was carried out using the standard analytical procedures previously described by Evans [26].

### Quantitative determination of total phenolic content (TPC)

The concentration of total phenolics in each plant extract was determined according to the method described by El-Hashash et al. [27]. Briefly, a mixture of 100 µl of plant extract (100 µg ml^–1^), 500 µl of Folin-Ciocalteu reagent, and 1.5 ml of Na_2_CO_3_ (20%) was shaken and diluted up to 10 ml with water. After two hours, the absorbance was measured at 765 nm. All determinations were carried out in triplicate. Gallic acid was standard, and the total phenol contents were expressed as milligrams of gallic acid equivalents per gram dry weight of the extract (mg GAE/g DW).

### Quantitative determination of total flavonoid contents (TFC)

The total flavonoid content was determined using the method described by El-Hashash et al. [27]. Briefly, 100 µl of 20% aluminum trichloride (AlCl_3_) in methanol was mixed with the same volume of extract solution (10 mg/ml) and a drop of acetic acid. The mixture was diluted up to 5 mL with methanol. The absorption was carried out at 415 nm using a spectrophotometer (UV–Vis spectrophotometer, Milton Roy 601), and readings were taken after 40 min against a blank sample. All determinations were carried out in triplicate. The total flavonoid content was expressed as mg rutin equivalents per gram dry weight of extract (mg RE/g DW).

### LC–ESI–MS analysis of C. rotundus 90% MeOH extract

In the negative ion mode, the 90% MeOH extract of *C. rotundus* was chemo-profiled using liquid chromatography coupled with electrospray ionization mass spectrometry (LC–ESI–MS). Experiments were performed on the LC system (Waters Alliance 2695, Waters, USA), using reversed-phase column C18, 250 mm, and 5 μm particle size (Phenomenex, USA). Eluent A was H_2_O acidified with 0.1% formic acid, and eluent B was CH_3_CN: MeOH (1:1), acidified with 0.1% formic acid. The injection volume was 20 μl of a 5 mg/ml 90% MeOH extract, and the elution flow was 400 μl/min. The program was the following: 0.0–5.0 min (5% B), 5.0–10 min (5.0%–10% B), 10–55 min (10%–50% B), 55–65 min (50%–95% B), 65–70 min (5% B). The ESI–MS spectra were estimated by scanning in the range of 50–1000 m/z with these parameters: the source temperature was set at 150 C, the cone voltage was 50 eV, the capillary voltage was 3 kV, the desolvation gas flow was 600 L/hour, the desolvation temperature was 350 C, and the cone gas flow was 50 L/hour. The compounds were assigned by retention time, and mass spectroscopic results were compared to standards and literature data.

### Animals and parasites

The present study was performed on white albino male mice of the CDI strain (aged 4–6 weeks and weighing 20–25 g) obtained from the TBRI biological unit. The mice were maintained hygienically throughout the study and fed regular commercial pelleted food with water as needed. Following approval from the Research Ethics Committee of TBRI (REC-TBRI), all animal experiments were carried out following guidelines recognized internationally.

The *Trichinella spiralis* (code: ISS6158) used in this study was provided by the Medical Parasitology Department, Tanta Faculty of Medicine, and was kept in the Parasitology Department, TBRI, by consecutive passages on mice and rats. Mice were orally infected with 200–300 T*. spiralis* larvae [28, 29].

### T. spiralis adult worms and muscle larvae isolation

According to ozkoc et al. [30], *T. spiralis* adults and muscle larvae were recovered from infected mice. The muscles of white albino mice infected with *T. spiralis* for 30 days were removed and minced, and the larvae in the muscles were then put in an acid-pepsin solution [31]. An electric stirrer was used to mix the mixture at 37 C for 2 h [32]. The mixture was filtered based on Kapel et al. [33]. The collected larvae were cleaned two to three times using tap water before being suspended in a conical flask for half an hour to permit sedimentation. The adult worms of *T. spiralis* were recovered from the small intestines of infected mice on the sixth-day post-infection (p.i).

To allow the worms to move out of the tissue, the intestine was cleaned, longitudinally opened along its entire length, sliced into parts measuring 2 cm, and then submerged in normal saline for 3–4 h at 37 C [34].

### *Design of *in vitro* and *in vivo* experiments*

On a 24-well tissue culture plate, adult *T. spiralis* worms (25 parasites per well) were grown using RPMI-1640 media as the incubation medium, which contained 20% fetal bovine serum, 200 U/mL penicillin, and 200 g/ml streptomycin.

The range of concentrations of each *C. rotundus* extract against adults was 25 to 500 µg/mL [24], while the range of ABZ against adults was 25 to 400 µg/mL [35]. Parasite controls and dimethyl sulfoxide (DMSO) controls were set, and each determination was performed in duplicate. The plates were incubated at 37 C with 5% CO_2_ for 1, 4, 24, 48, and 72 h. At the end of the incubation periods, the parasites (both dead and living) in the wells were counted by inverted microscopy. The adult motility assay is the method of choice to evaluate the drug sensitivity of different nematode species. Non-motile worms were considered dead, and the survival rate in each well was calculated [36]. Worm mortality % = (the number of dead worms / the total number of worms) × 100%. The criteria for a dead body were that the worm’s body was C-shaped or linear, and there was no movement [37]. Then, adult worms were gathered for scanning electron microscopy examination.

For the in vivo study, mice were distributed into five groups (*n* = 12):

- Group I: non-infected and untreated (control-negative).
- Group II: infected but not treated (control-positive).
- Group III: infected and treated with ABZ (pure powder was purchased from Sigma-Aldrich, St. Louis, MO, USA) given at a 50 mg/kg dose orally [38].
- Group IV: infected and treated with the most promising *Cyperus rotundus* extract in the in vitro studies (given orally at 250 mg/kg) [39].
- Group V: infected and treated with a combination of ABZ (given at a 50 mg/kg dose orally) and *Cyperus rotundus* extract (given orally at 250 mg/kg).

To evaluate the effects of the medications administered during the intestinal phase (a) (2–6 days p.i) and the muscular phase (30–34 days p.i) individually, groups II–V were divided into two subgroups (a and b) (*n* = 6).

### Determination of the burden of adult worms and muscle larvae of T. spiralis

In subgroup (a), the mice were sacrificed on day 7 p.i. under light anesthesia by isoflurane inhalation (Forane®, Baxter, UK) to assess the effects of therapy on the intestinal phase, and the small intestine was processed as previously reported [34]. Adults of *T. spiralis* were collected and counted, and the reduction rate of worms was calculated.

On day 35 after infection, the mice in subgroup (b) were sacrificed under light anesthesia using isoflurane inhalation, and the muscle larvae were recovered using the pepsin digestion procedure. This was carried out to examine the effects of the therapy on the muscular phase.

The collected larvae were counted microscopically using a McMaster counting chamber. The number of larvae per gram of digested carcass (Muscle larvae/g) (ML/g) served as a measure of parasite burdens [40, 41].

### Scanning electron microscopy

Adult worms were handled using the methods outlined by Abou Rayia et al. [29]. A fresh fixation solution containing 2.5% glutaraldehyde solution buffered with 0.1 M sodium cacodylate at pH 7.2 was used to fix worms from each group immediately and kept overnight at 4 C. After that, the fixed specimens were washed in 0.1 M sodium cacodylate buffer at pH 7.2 for 5 min, post-fixed in 2% osmium tetroxide for 1 h, and rinsed in distilled water.

The samples were dehydrated in ethyl alcohol in increasing concentrations, mounted on adhesive material with a carbon coating, and analyzed by FEI-Philipps scanning electron microscope [42, 43].

### Histopathological examination

Skeletal muscle and small intestine segments from the study groups were fixed in 10% formalin for 24 h, cleaned in water for 12 h, dehydrated in increasing grades of alcohol, and cleared in xylene. Pure soft paraffin was used for impregnation for two hours at a temperature of 55 C. Then, stiff paraffin sections were cut using a microtome at 5 μm. Hematoxylin and eosin stains were used to stain the sections [44].

### Statistical analysis

The data were analyzed using Microsoft Excel 2016 and a statistical package for social science (IBM SPSS Statistics for Windows, version 26, IBM Corp., Armonk, NY, USA). Continuous normally distributed variables were represented as mean ± SD, with a 95% confidence interval. The Student’s *t*-test was performed to compare the means of normally distributed variables between groups, besides ANOVA and Dunnett T3 as post hoc tests in multigroups. *P*-value < 0.05 was significant, and *P*-value < 0.001 was highly significant [45].

## Results

### Phytochemical screening and total phenolic and flavonoid contents of C. rotundus extracts

The phytochemical screenings were carried out using standardized lab protocols to investigate the secondary metabolites of *C. rotundus* 90% MeOH ext. and their derived fractions (EtOAc fr., pet-ether fr., and n-BuOH fr.). The results showed that they contained several groups of active ingredients (Table 1). The 90% MeOH ext., EtOAc fr., and n-BuOH fr. showed a high number of flavonoids and phenols. Moreover, the 90% MeOH ext. and pet-ether fr. demonstrated a high content of terpenoids and sterols. However, the concentrations of the other phytoconstituents, such as alkaloids, tannins, glycosides, and saponins, ranged from none to moderate amounts in the 90% MeOH ext., and their fractions.

**Table 1:** Preliminary phytochemical screening of *C. rotundus* extracts

**Constituents**	**90% MeOH ext**	**Pet-ether fr**	**EtOAc fr**	n-**BuOH fr**
**Flavonoids**	+ + +	+	+ + +	+ + +
**Alkaloids**	+ +	-	+	+ +
**Tannins**	+	-	+	+
**Sterols**	+ + +	+ + +	+	+
**Terpenoids**	+ + +	+ + +	+ +	+ + +
**Glycosides**	+ +	+ +	+ +	+ +
**Phenols**	+ + +	+	+ + +	+ + +
**Saponins**	+ +	+	+	+ +

(+ + +): high amount

(+ +): moderate amount

( +): small amount

(-): Absent

The distribution of phenolic and flavonoid compounds in *C. rotundus* extracts (Table 2) demonstrated that the EtOAc fr. had the highest number of phenols and flavonoids with values of 567.35 ± 7.89 mg GAE/g DW and 316.32 ± 2.59 mg RE/g DW, respectively, followed by n-BuOH fr. (277.40 ± 4.46 mg GAE/g DW and 165.38 ± 2.45 mg RE/g DW, respectively), 90% MeOH ext. (174.66 ± 2.35 mg GAE/g DW, and 121.71 ± 1.67 mg RE/g DW, respectively). The lowest phenolic content was observed in pet-ether fr. with values of 97.03 ± 2.03 mg GAE/g DW and 106.26 ± 0.50 mg RE/g DW, respectively.

**Table 2:** Total phenolic and flavonoid contents of *C. rotundus* extracts

Extract	Total phenols (mg GAE/g dry extract)	Total flavonoids (mg RE/g dry extract)
**90% MeOH ext**	174.66 ± 2.35	121.71 ± 1.67
**Pet-ether fr**	97.03 ± 2.03	106.26 ± 0.50
**EtOAc fr**	567.35 ± 7.89	316.32 ± 2.59
**n-BuOH fr**	277.40 ± 4.46	165.38 ± 2.45

Results are expressed as a mean of triplicate determinations ± SD

### LC–ESI–MS characterization of the primary phytochemicals of C. rotundus 90% MeOH ext

**Fig. 2:**
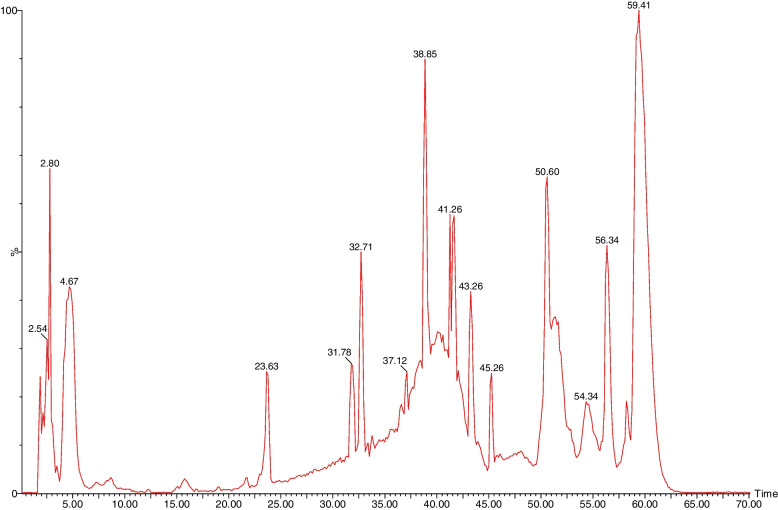
Total ion chromatogram (TIC) of *C. rotundus* 90% MeOH extract

**Fig. 3:**
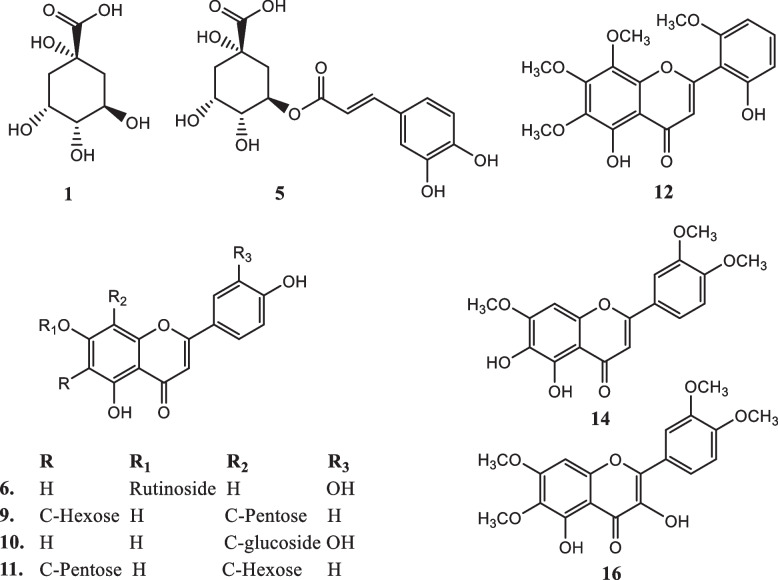
Structures of some detected compounds in the 90% MeOH extract of *C. rotundus*

The phenolic compounds from *C. rotundus* 90% MeOH ext. have been obtained using LC–ESI–MS in negative ionization mode. Twenty-six different phenolic compounds were tentatively assigned in *C. rotundus* 90% MeOH ext., which included five phenolic acids, 18 flavonoids, one organic acid, and two unknown phenolic compounds, as mentioned in Table 3. The total ion chromatogram of the identified compounds is represented in Fig. 2, and the chemical structures of some detected compounds are shown in Fig. 3. Additionally, the chemical components were identified by comparing MS fragmentation patterns and molecular ion peaks with the literature.

**Table 3:** Tentative assignment of chemical constituents of 90% MeOH extract of *C. rotundus* by LC-ESI -MS

Comp. No	t_R_ (min)	MW	[M-H]^−^	*m/z* fragments	Tentative assignment
1	12.28	192	191	173	Quinic acid
2	15.09	330	329	315, 203, 116	Unknown
3	16.96	388	387	211, 197	Trimethyl-gallic acid-glucuronide
4	17.76	330	329	314, 135	Trihydroxy-octadecanoic acid
5	18.96	354	353	191 (100%), 179 (63%), 135	3-O- caffeoylquinic acid
6	21.63	594	593	285, 175, 151	Luteolin-7*-*O-rutinoside
7	23.10	354	353	191 (100%), 179 (13%), 135	4-O- caffeoylquinic acid
8	23.77	354	353	191 (100%), 179, 161	5-O- caffeoylquinic acid
9	31.91	564	563	473, 443, 383, 353	Apigenin-6-C-hexoside-8-C-pentoside
10	32.71	448	447	357, 327, 297	Luteolin-8-C-glucoside (Orientin)
11	33.78	564	563	503, 473, 443	Isoschaftoside
12	36.58	374	373	343, 328, 300	Skullcapflavone II
13	37.12	594	593	447, 285, 175	Luteolin-7-O-rutinoside
14	37.65	344	343	328, 285, 269, 313	5,6-dihydroxy-3’,4’,7-trimethyl-flavone
15	38.85	462	461	285	Luteolin-7-O-glucoronoid
16	40.19	374	373	358, 343, 328, 313, 269	Quercetagetin-tetramethyl ester
17	41.26	638	637	491, 329, 313	Tricin-O-rutinoside
18	41.66	654	653	491, 329, 313	Tricin-O-dihexosides
19	42.06	374	373	358, 343, 328, 313, 269	Quercetagetin-tetramethyl ester isomer
20	43.26	638	637	505, 329, 314	Tricin-O-ferulyl-pentoside
21	45.26	834	833	637, 359, 301, 269	Unknown
22	50.60	286	285	199, 175, 151, 133	Luteolin
23	51.40	374	373	358, 343, 328	Quercetagetin-tetramethyl ester isomer
24	54.48	620	619	577, 431, 269, 175	Apigenin-7-O-Acetyl-rutinoside
25	56.35	820	819	741, 619, 577, 269, 175	Apigenin derivatives
26	59.42	344	343	313, 298, 270	5,6-dihydroxy-3’,4’,7-trimethoxy flavone isomer

*t*_*R*_ retention time, *M.W* molecular weight, *[M-H]* deprotonated molecular ion

### In vitro* anthelmintic activity*

Survival numbers of *T. spiralis* adult worms incubated with different concentrations of compounds under study by exposure times are shown in Table 4. The effect of different compounds being studied on *T. spiralis* adult worms’ survival depended on concentration and time.

**Table 4:** Survival numbers of *T. spiralis* adult worms incubated with different concentrations of *C. rotundus* 90% MeOH extract and its derived fractions by exposure times (*n* = 25)

Drugs	Drug dose μg/ml	Exposure time (h)					LC_50_*μ*g/ml
		1 h	4 h	24 h	48 h	72 h	
Parasite control		25 ± 0.0	25 ± 0.0	23 ± 0.2	22 ± 0.35	20.5 ± 0.41	
DMSO control		24.5 ± 0.02	24 ± 0.1	22.5 ± 0.35	20.5 ± 0.8	20 ± 0.95	
Albendazole	400	4 ± 0.93**	0 ± 0**	0 ± 0**	0 ± 0**	0 ± 0**	71.41
	200	11.5 ± 2.5**	8 ± 2.8**	0 ± 0**	0 ± 0**	0 ± 0**	
	100	19.5 ± 3.2*	16 ± 2.65*	6.5 ± 1.23**	0 ± 0**	0 ± 0**	
	50	23.5 ± 1.8	20.5 ± 2.3	17.5 ± 3.5*	11.5 ± 3.5*	9.5 ± 2.5*	
	25	24.5 ± 3.4	23 ± 2.5	20.5 ± 3.5	17.5 ± 3.7	15 ± 2.8	
90%MeOH ext	500	19.5 ± 3.2*	18.5 ± 4.3*	12 ± 3.5*	6 ± 1.3**	0 ± 0**	156.13
	250	21.5 ± 4.2	20 ± 3.2	16.5 ± 2.4*	9.5 ± 2.3**	2.5 ± 3.5**	
	125	23.5 ± 3.9	23 ± 5.2	19.5 ± 3.6	17 ± 1.5	11.5 ± 1.5*	
	50	25 ± 5.4	24.5 ± 3.1	22.5 ± 2.8	19.5 ± 2.9	17 ± 2.5	
	25	25 ± 3.8	25 ± 4.2	23.5 ± 3.5	20.5 ± 3.6	20.5 ± 2.3	
EtOAc fr	500	17.5 ± 2.8*	15 ± 3.4*	8 ± 2.5**	3.5 ± 1.2**	0 ± 0**	294.67
	250	20.5 ± 3.8	18 ± 2.1*	13.5 ± 3.6*	7 ± 2.0**	0 ± 0**	
	125	22.5 ± 2.9	22 ± 3.6	16.5 ± 2.4*	13 ± 3.2*	8.5 ± 2.1**	
	50	23.5 ± 3.7	23 ± 2.7	21 ± 2.1	18.5 ± 2.5	16 ± 1.2	
	25	25 ± 2.8	24 ± 4.2	23.5 ± 3.5	21 ± 3.1	20 ± 2.3	
Pet-ether fr	500	12.5 ± 3.9**	9 ± 3.1**	3.0 ± 0.9**	0 ± 0**	0 ± 0**	82.09
	250	18.5 ± 2.7*	15 ± 4.1*	9.5 ± 2.5**	2.5 ± 0.9**	0 ± 0**	
	125	22.5 ± 3.2	18 ± 3.1*	14.5 ± 1.6*	8.5 ± 1.6**	3 ± 1.2**	
	50	24.5 ± 4.2	23 ± 2.5	18.5 ± 3.2	13.5 ± 3.2	12.5 ± 2.3*	
	25	25 ± 1.9	23.5 ± 3.5	22.5 ± 2.1	20.5 ± 2.5	19.5 ± 3.1	
*n*-BuOH fr	500	9.5 ± 3.5**	6 ± 3.6**	0 ± 0**	0 ± 0**	0 ± 0**	73.16
	250	14.5 ± 4.3**	10 ± 2.4**	2.5 ± .2**	0 ± 0**	0 ± 0**	
	125	19.5 ± 2.3*	16 ± 1.8*	10.5 ± 3.2**	0 ± 0**	0 ± 0**	
	50	23 ± 4.3	22.5 ± 6.8	18 ± 2.5	14 ± 3.2	11 ± 3.2*	
	25	24 ± 2.9	23.5 ± 3.3	21.5 ± 1.9	18.5 ± 2.1	17.5 ± 2.4	

The survival of adult worms was represented as mean ± SD. The data were analyzed by Student’s *t*-test

^*^P value < 0.05 was significant

^**^P value < 0.01 was highly significant

LC_50_ were calculated based on the Quest Graph online program: https://www.aatbio.com/tools/ic50-calculator. Each well/each plate contained 25 living adult worms at the beginning of the in vitro cultivation

Albendazole caused the death of all adult worms after 48 h at concentrations starting from 100 µg/ml. The statistically significant effect of ABZ was evident from the first hour of incubation at a concentration of 100 µg/ml. The LC_50_ of ABZ was calculated to be 71.41 µg/ml.

Regarding *C. rotundus*’ different extracts, the 90% MeOH ext. caused the death of all adult worms at the highest concentration (500 µg/ml) after 72 h. The same activity was recorded with EtOAc fr. at high concentrations (250 µg/ml and 500 µg/ml) after 72 h, pet-ether fr. at the highest concentration (500 µg/ml) after 48 h, and n-BuOH fr. after 48 h at concentrations starting from 125 µg/ml.

A statistically significant difference was recorded initially with 90% MeOH ext. after incubation at 125 µg/ml for 72 h. While in EtOAc fr., it was determined after incubation at 125 µg/ml for 24 h. It was reported with pet-ether fr. after incubation at 50 µg/ml for 72 h. The statistically significant effect of n-BuOH fr. was evident from the first hour of incubation in a 125 µg/ml concentration.

The LC_50_ was calculated to be 156.12 µg/ml, 294.67 µg/ml, 82.09 µg/ml, and 73.16 µg/ml in 90% MeOH ext., EtOAc fr., pet-ether fr., and n-BuOH fr., respectively.

There was no significant difference between parasite controls and DMSO controls at all test incubation periods.

### Mortality rates of T. spiralis adult worms exposed to different concentrations of C. rotundus 90% MeOH ext. and its derived fractions by exposure times

Table 5 shows the mortality rates of *T. spiralis* adult worms following incubation with different concentrations of the drugs under study by exposure times.

**Table 5:** Mortality rates (%) of *T. spiralis* adult worms exposed to different concentrations of *C. rotundus* 90% MeOH extract and its derived fractions by exposure times

Mortality rates (%)	Drug dose *μ*g/ml	Exposure time (h)				
Drugs		1 h	4 h	24 h	48 h	72 h
Albendazole	400	84	100	100	100	100
	200	54	68	100	100	100
	100	22	36	74	100	100
	50	6	18	30	54	62
	25	2	8	18	30	40
90% MeOH ext	500	22	26	52	76	100
	250	14	20	34	62	90
	125	6	8	22	32	54
	50	0	2	10	22	32
	25	0	0	6	18	18
EtOAc fr	500	30	40	68	86	100
	250	18	28	46	72	100
	125	10	12	34	48	66
	50	6	8	16	26	36
	25	0	4	6	16	20
Pet-ether fr	500	50	64	88	100	100
	250	26	40	62	90	100
	125	10	28	42	66	88
	50	2	8	26	46	50
	25	0	6	10	18	22
*n*-BuOH fr	500	62	76	100	100	100
	250	42	60	90	100	100
	125	22	36	58	100	100
	50	8	10	28	44	56
	25	4	6	14	26	30

Worm mortality % = (the number of dead worms / total number of worms) × 100%

Incubation of adult worms in ABZ drug resulted in increased mortality rates after 4 h to 18% at 50 µg/ml. Increasing the concentration to 100 µg/ml increased the mortality rate to 36% after 4 h. Furthermore, ABZ killed all adult worms after 48 h at 100 µg/ml and after 24 h at 200 µg/ml.

Concerning *C. rotundus* different extracts, the mortality rates increased after 4 h of incubation at 125 µg/ml in 90% MeOH ext, EtOAc fr., pet-ether fr. and n-BuOH fr. to 8%, 12%, 28%, and 36%, respectively. Additionally, mortality rates increased to 22%, 34%, 42%, and 58% after 24 h at the same concentration in 90% MeOH ext, EtOAc fr., pet-ether fr., and n-BuOH fr., respectively.

Regarding *C. rotundus* different extracts, 90% MeOH extract killed all adult worms at the highest concentration (500 µg/ml) after 72 h. The same action was recorded with EtOAc fr. at high concentrations (250 µg/ml and 500 µg/ml) after 72 h, pet-ether fr. at the highest concentration (500 µg/ml) after 48 h, and n-BuOH fr. after 48 h at concentrations starting from 125 µg/ml.

### Scanning electron microscope (SEM) findings

**Fig. 4:**
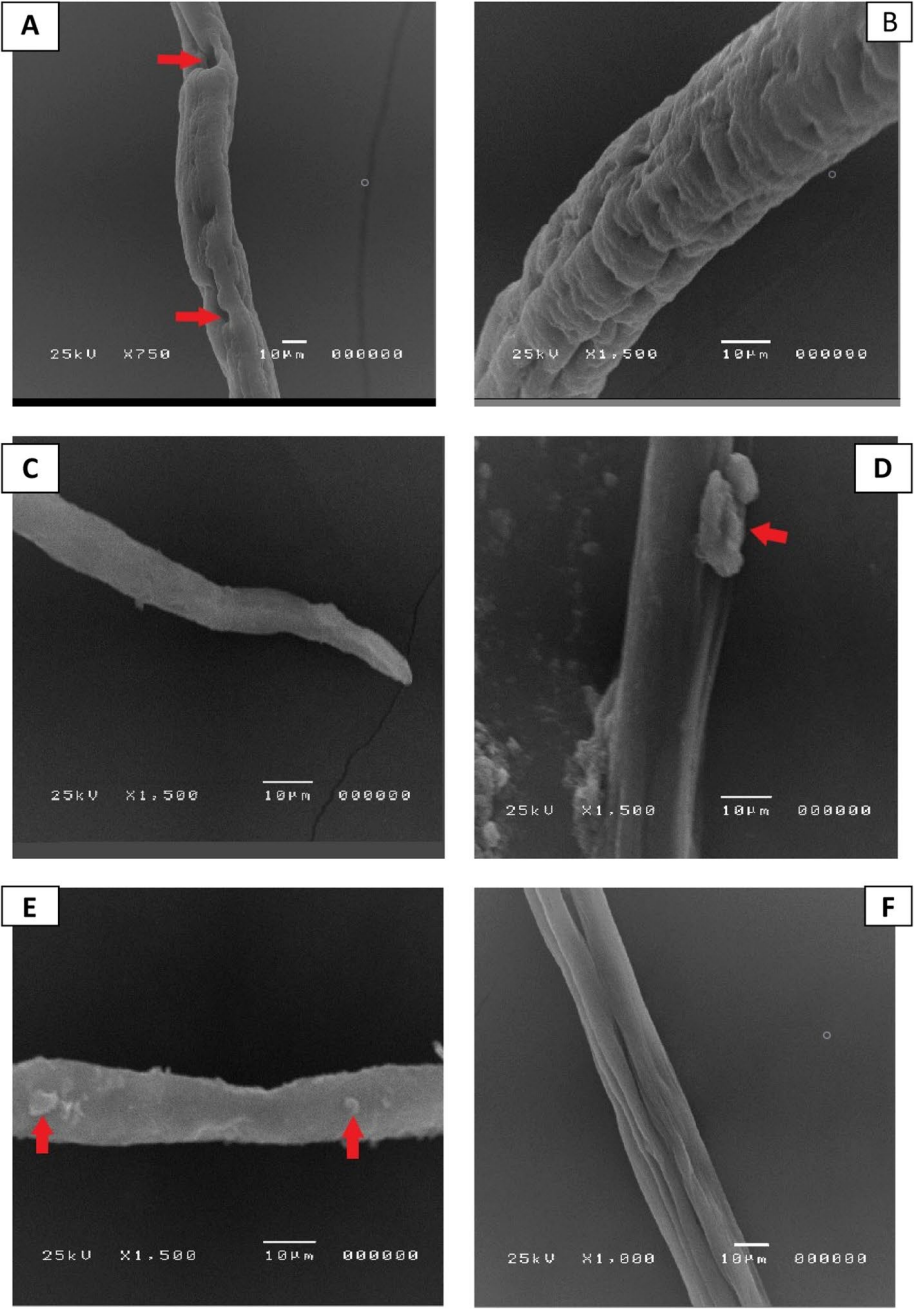
SEM results of adult *T. spiralis* in culture: **A**, **B** showing typical worm, **C**, **D** demonstrating group that received albendazole with severe destruction of the adult worm, normal crease loss, and appearance of cauliflower masses (red arrows), and **E**, **F** groups that received n-BuOH fraction with remarkable adult worm destruction, annulation loss, and appearance of blebs and vesicles (red arrows)

Regarding the adult worm’s morphology in the control group (incubated in culture media or DMSO without drugs), the cuticle retained the typical structure as annulations, ridges, transverse creases, and hypodermal gland openings (Fig. 4A, B).

In the ABZ-treated group, marked destruction of the adult worms was obvious. There was a loss of annulations, loss of the normal creases, and appearance of cauliflower masses (Fig. 4C, D).

Regarding *C. rotundus*, different extracts, including n-BuOH fr. proved to have the most promising effects in in vitro studies. As a result, the n-BuOH fr. treated group was subjected to SEM examination. The loss of the normal morphology was observed in the n-BuOH fr., and the treated group lost the typical creases, annulations, and formation of areas containing blebs and vesicles (Fig. 4E, F).

### In vivo* results*

#### Count of the adult worms in the small intestine

When compared to the control group (105.8 ± 7.3), the mean number of adult worms significantly (*P* < 0.001) decreased in all treatment groups. The lowest mean adult worm count was witnessed in GVa**, **which received combination therapy (9.8 ± 1.9) and showed the highest elimination of *T. spiralis* adult worms with (91%) efficacy, followed by GIIIa, which received ABZ (12.8 ± 3.2) with 88% efficacy. While in GIVa, which received n-BuOH fr., the mean adult worm count was 32.6 ± 3.487 with a reasonable percentage of reduction (70%) (Table 6).

**Table 6:** Adult worm count of *Trichinella spiralis* in the small intestine at seven days post-infection

Intestinal phase		GII: Control-Positive	GIII: Abz-treated	GIV: CR n-BuOH fr. treated	GV: Com of Abz + CR n-BuOH fr
**Mean ± SD**		105.8 ± 7.3	12.8 ± 3.2	32.6 ± 3.4	9.8 ± 1.9
^**a**^***P*-value**		-	0.001**	0.001**	0.001**
**ANOVA**		^**b**^***P*-value** = 0.001**			
**Post hoc test** **Dunnett T3** ^**c**^***P*-value**	**GII:**	-	0.001**	0.001**	0.001**
	**GIII:**	-	-	0.001**	0.4
	**GIV:**	-	-	-	0.001**
	**GV:**	-	-	-	-
**% of Efficacy**			88%	70%	91%

*T. spiralis* adult worm count was represented as mean ± SD. In contrast, the efficacy was represented as a percentage based on the following low %: Efficacy = [(Mean count in the infected control group − Mean count in the study group) / Mean count in the infected control group] × 100

^a^*p*-value is significantly different from the control based on the Student’s t-test

^b^*p*-value is significantly different when comparing groups based on the one-way ANOVA test

^c^*p*-value significantly differs between groups depending on a post hoc test (Dunnett T3)

^*^p-value < 0.05 is significant

^**^p-value < 0.01 is highly significant

#### Count of encysted larvae in muscles

Regarding the effects of a drug on muscle phase, there was a significant decrease (*P* < 0.001) in the mean larval count per gram muscle in all treated groups when compared with the control group (3520.0 ± 432.4).

The highest mean larval count reduction was detected in group GVb, which received the combination therapy (682.0 ± 34.2) with 81% efficacy, followed by the ABZ-treated group (GIIIb) (839.6 ± 49.8) with 76% efficacy. In the n-BuOH fr., treated group (GIVb), the mean larval count was 1236.0 ± 68.8 with a 65% reduction percentage (Table 7).

**Table 7:** Count of *Trichinella spiralis* encysted larvae per gram muscle at 35 days post-infection

Muscular phase		GII: Control-Positive	GIII: Abz-treated	GIV: CR n-BuOH fr. treated	GV: Com of Abz + CR n-BuOH fr
**Mean ± SD**		3520.0 ± 432.4	839.6 ± 49.8	1236.0 ± 68.8	682.0 ± 34.2
^**a**^***P*-value**		-	0.001**	0.001**	0.001**
**ANOVA**		^**b**^***P***-**value** = 0.001**			
**Post hoc test** **Dunnett T3** ^**c**^***P*-value**	**GII:**	-	0.001**	0.001**	0.001**
	**GIII:**	-	-	0.001**	0.003**
	**GIV:**	-	-	-	0.001**
	**GV:**	-	-	-	-
**% of Efficacy**			76%	65%	81%

*T. spiralis* larvae count was represented as mean ± SD, while the efficacy was represented as percentage depending on the following low %:

Efficacy = [(Mean count in the infected control group − Mean count in the study group) / Mean count in the infected control group] × 100

^a^*p*-value is significantly different from the control based on the Student’s t-test

^b^*p*-value is significantly different when comparing groups based on the one-way ANOVA test

^c^*p*-value significantly differs between groups depending on a post hoc test (Dunnett T3)

^*^p-value < 0.05 is significant

^**^p-value < 0.01 is highly significant

### Histopathological finding

#### Small intestine examination

**Fig. 5:**
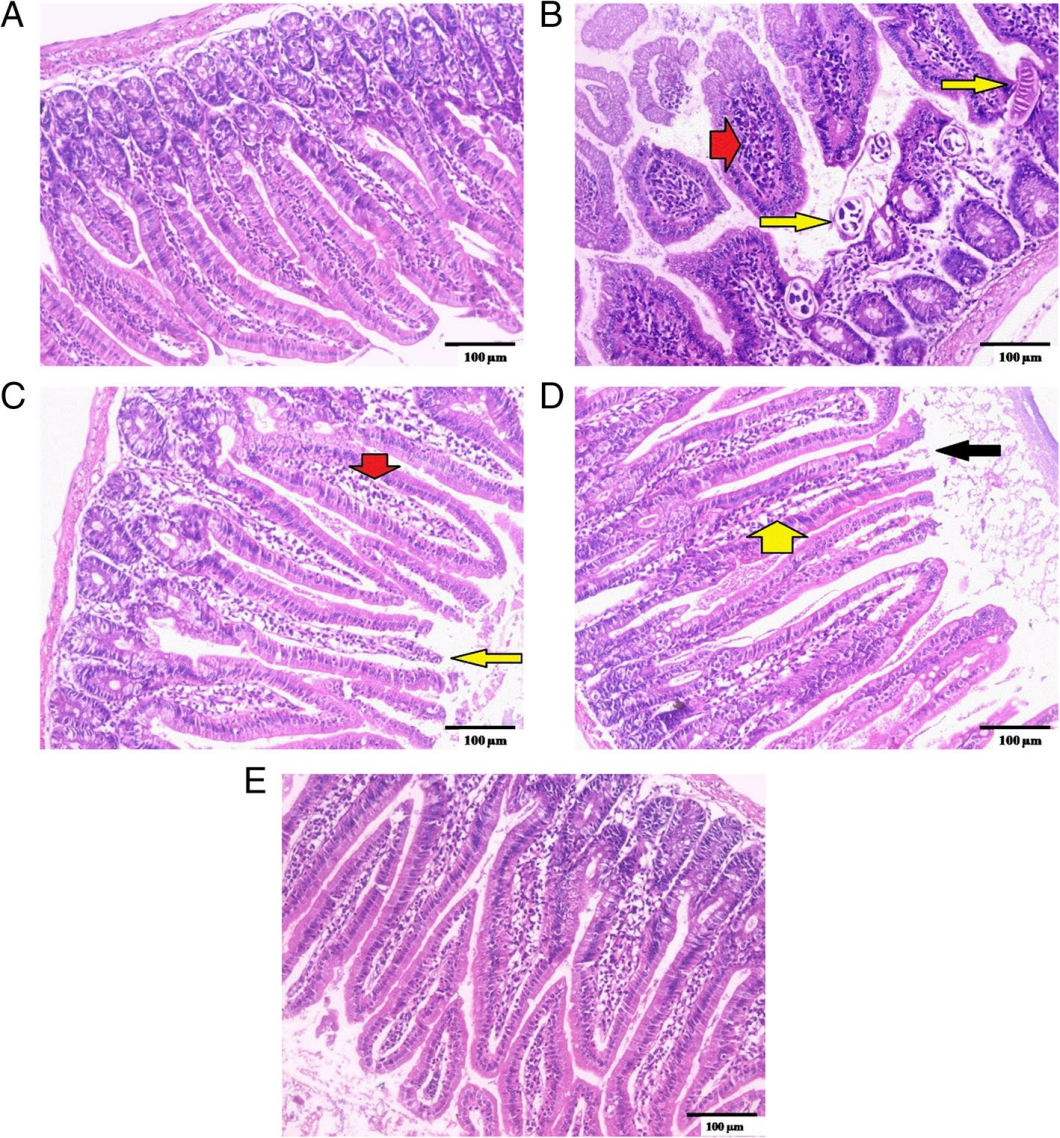
Histopathological examination of sections from the small intestine: **A** GI, normal control mice (uninfected), showing a normal villous pattern (H&E, X200). **B** GII: positive control mice (infected), showing an irregular villous pattern, with villous expansion by inflammatory cells (red arrow) and scattered fragments of the worms (yellow arrows) (H&E, X200). **C** Infected mice treated with ABZ showed mostly regular villous patterns, with focal villous tip erosion (yellow arrow) and mild inflammation (red arrow) (H&E, X200). **D** Infected mice treated with n-BuOH fr. showed a focally irregular villous pattern, with villous tip erosion (black arrow) and mild inflammation (yellow arrow) (H&E, X200). **E** Infected mice treated with combination therapy showed a nearly restored regular villous pattern with fewer inflammatory cells (H&E, X200)

Histopathological examination of small intestine sections from the standard control group (GI) demonstrated typical architecture with healthy mucosa and average crypts/villous ratio. Goblet cells were moderate in number with a well-defined brush border and lamina propria (Fig. 5A).

The intestinal wall of the infected control group (GII) showed abundant intervillous inflammatory cellular infiltration that was primarily composed of lymphocytes and plasma cells, which are mononuclear cells. The intestinal villi showed broadening and atrophy. Additionally, adult worm fragments were found within the intestinal lumen (Fig. 5B).

According to the results of the sections from the examined treated groups (Fig. 5C, D, and E), an obvious reduction in the severity of the inflammatory cellular infiltration was witnessed, along with a striking improvement in the other histopathological changes of the intestine and the return of the regular villous pattern in GVa (Fig. 5E).

#### Skeletal muscle examination

**Fig. 6:**
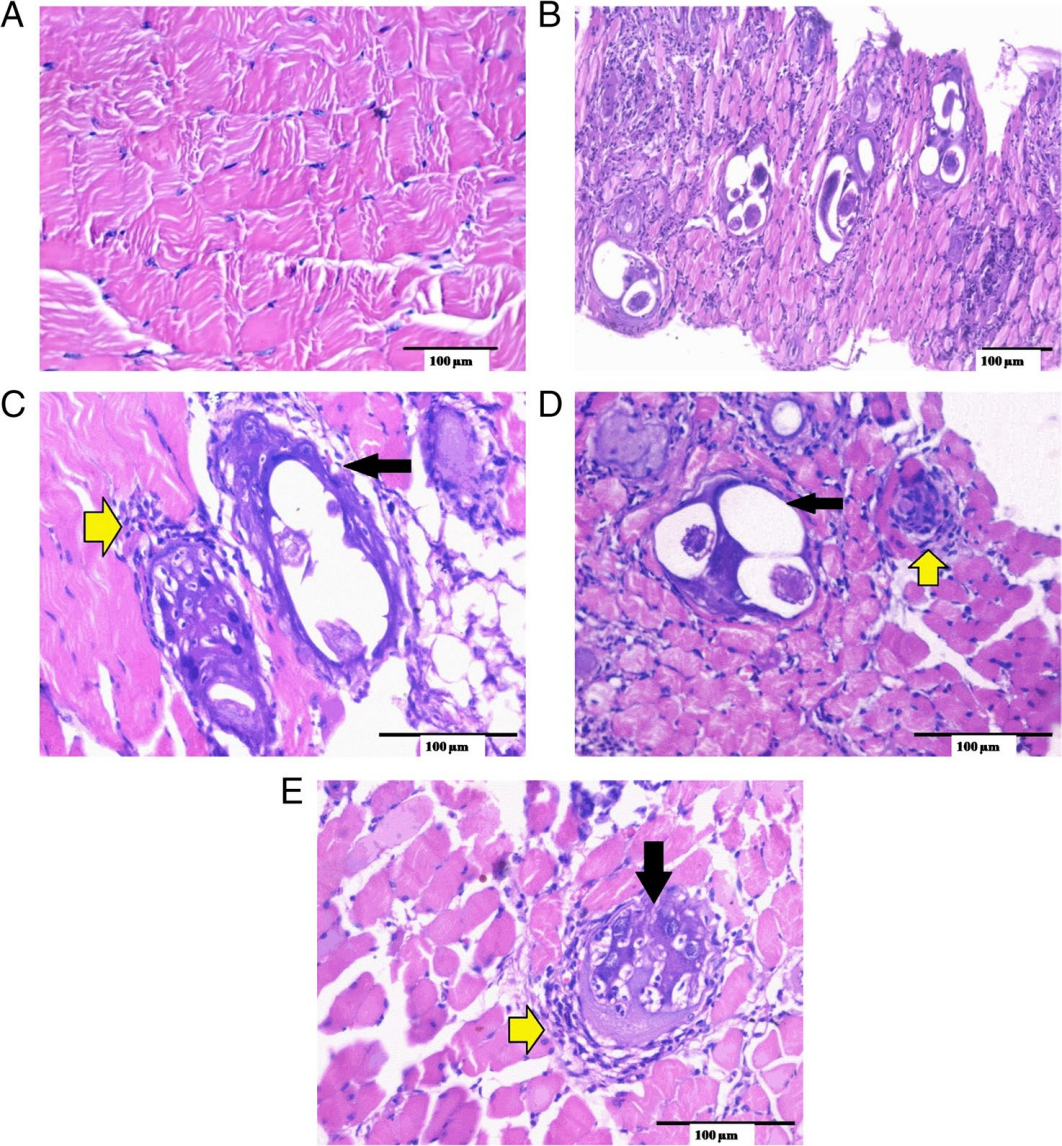
Histopathological examination of muscular sections: **A** GI, normal control mice (uninfected) showed normal skeletal muscle pattern (H&E, X200). **B** GII: positive control mice (infected) showed several cysts and a moderate inflammatory cellular reaction (yellow arrows) (H&E, X200). **c** Infected mice treated with ABZ showed remnants of cysts, with degenerated capsules (black arrow) and contents surrounded by many macrophages (yellow arrow) (H&E, X400). **D** Infected mice treated with n-BuOH fr. demonstrated many cysts with degenerated capsules (black arrow) and contents surrounded by many macrophages (yellow arrow) (H&E, X400). **E** Infected mice treated with combination therapy demonstrated a single cyst with degenerated capsule and content (black arrow) surrounded and infiltrated by many macrophages (yellow arrow) (H&E, X400)

Histopathological examination of sections from muscles of the normal control group (GI) showed a normal skeletal muscle pattern (Fig. 6A). The infected control group (GI) demonstrated the presence of numerous encysted *T. spiralis* larvae dispersed throughout the muscle sarcoplasm and several chronic inflammatory cells in the form of lymphocytes, plasma cells, and histiocytes, infiltrating muscle bundles and surrounding the encysted larvae (Fig. 6B).

Examination of muscular sections from the treated groups (Fig. 6 C, D, and E) showed a remarkable improvement in the histopathological findings compared to GII, the infected control group. GIIIb and GIVb revealed a reduced number of cysts with degenerated capsules and localized pericapsular plasma-lymphocytic inflammatory cellular infiltration (Fig. 6C & D). While GVb demonstrated the best improvement with the least number of cysts and degenerated larvae capsules. Additionally, the size of the larvae was reduced, and their internal structure was destroyed (Fig. 6E).

## Discussion

Since ancient times, medicinal plants have been crucial in developing powerful therapeutic substances. According to current estimates, 80% of people in developing nations still rely on folk medicine to treat various common health issues. Furthermore, herbal medications are in more significant demand than ever, and their acceptance has grown over time [46, 47].

The pharmacological treatment of trichinellosis is debatable. Albendazole is one of the benzimidazoles and is the drug of choice in trichinellosis treatment. However, albendazole has been linked to various adverse medication responses, including fatalities, encephalitis, seizures, and severe drug eruptions [6, 48].

Additionally, it exhibits a weak susceptibility to migrating and encapsulated muscle larvae [49]. That might explain the urgent need for a new, secure, potent therapy to eradicate *Trichinella* spp. infection.

*Cyperus rotundus*, a worldwide herb used in conventional medicine to treat several diseases, is regarded as a plant with infinite medicinal properties validated by the scientific committee [12, 50, 51]. Moreover, *C. rotundus* has a wide range of safety features. The researchers documented that administering *C. rotundus* extract orally in rats did not induce acute toxicity, and there was no mortality or behavior changes for subacute toxicity [52].

In the current study, the in vitro anti-trichinellosis potential of the active phytoconstituents of *C. rotundus* aerial part 90% methanol extract and its derived fractions (EtOAc fr., pet-ether fr., and n-BuOH fr.) were determined. The fraction with the most promising effects was then used to evaluate the in vivo therapeutic effects of *C. rotundus*.

The preliminary phytochemical screening tests are valuable for investigating the bioactive plant secondary metabolites [53]. *C. rotundus* 90% MeOH extract, and its derived fractions included high quantities of flavonoids, phenols, sterols, and triterpenoids, which had different pharmacological properties. The previous phytochemical surveys on the different parts of *C. rotundus* documented the presence of sesquiterpenes, phenylpropanoids, phenolics, alkaloids, flavonoids, and iridoids in rich amounts [12, 19]. These secondary metabolites played an insignificant role in the growth of the plant. However, they were essential for various defense mechanisms against the harmful effects of UV radiation, herbivore, and microbial attack [54].

Phenolics and flavonoids are the major groups of secondary metabolites, especially in plants, and have been considered responsible for various pharmacological activities [55]. Phenolic and flavonoids represent one of the most diverse groups of natural compounds. Therefore, the 90% MeOH ext. of *C. rotundus* and its derived fractions were analyzed for total phenolic and flavonoid contents in this study. The findings showed that *C. rotundus*' EtOAc fr. had a higher concentration of phenols and flavonoids than its n-BuOH fr., 90% MeOH ext., and pet-ether fr. It was reported that the concentration of phenolic compounds in plants depended on environmental factors such as light, temperature, and soil salinity. Furthermore, the solubility of phenolic compounds is governed by the kind of extraction and solvent polarity [56]. The previous reports stated that the 70% ethanol extract of dried rhizomes of *C. rotundus* had a total phenolic content value of 73.27 ± 4.26 mg catechin equivalents/g of dried rhizome extract [57]. In addition, the different extracts of *C. rotundus* (hexane, petroleum ether, ethyl acetate, chloroform, 70% acetone, 70% ethanol, 70% methanol, and water) were quantitatively analyzed for TPC and TFC. The TPC of the different extracts ranged from 0.0358 ± 0.002 to 118.924 ± 5.946 μg GAE/mg dry extract, and the TFC ranged from 7.196 ± 0.359 to 200.654 ± 10.032 μg quercetin equivalent (QE)/mg dry extract [58]. Other findings showed that the TPC of *C. rotundus* extracts (70% ethanol, MeOH, and water) ranged from 70.75 ± 4.48 to 254.50 ± 5.26 μg GAE/mg extract. In comparison, the TFC ranged from 51.23 ± 2.62 to 164.34 ± 3.75 μg catechin equivalents (CE)/mg extract [59]. Thus, our investigation proved that the Egyptian *C. rotundus* extracts had a significant number of phenolics and flavonoids, which could contribute to its promising medicinal properties.

The LC–ESI–MS analysis of the 90% MeOH extract of *C. rotundus* in negative ion mode revealed the presence of polyphenolic compounds, including phenolic acids, flavonoids (C-glycosyl and O-glycosyl), and organic acids. A detailed description of these identified compounds can be found in the Supplementary data file.

Moreover, to the best of our knowledge, some of these compounds were detected in *C. rotundus* for the first time, for example, compounds 3, 4, 9, 11, 12, 14, 16, 20, and 24, which encourages us to do further chromatographic isolation for these bioactive ingredients.

To save time and money, it is common practice to assess an agent’s potential anti-parasitic activity in vitro before trying in vivo research. It is not always the case that an agent’s in vivo activity will follow from its in vitro performance. This variance results from various factors, including the pharmacology and bioavailability of these drugs in the host [60].

An effective in vitro agent must be tested additionally in vivo. Therefore, we tested *C. rotundus* 90% MeOH ext. and its derived fractions (pet-ether fr. EtOAc fr., and n-BuOH fr.) in vitro. The in vitro studies proved that n-BuOH fr. had the most promising effects; as a result, the n-BuOH fr. treated group was subjected to SEM examination. Besides, in vivo testing was carried out for n-BuOH fr. using the murine model.

Regarding the in vitro studies for *C. rotundus* different extracts, the LC_50_ was calculated to be 156.12 µg/ml, 294.67 µg/ml, 82.09 µg/ml, and 73.16 µg/ml for 90% MeOH ext., EtOAc fr., pet-ether fr. and n-BuOH fr., respectively.

The in vitro or in vivo anti-parasitic activity of *C. rotundus* was documented in previous studies. The in vitro anthelmintic activity of *C. rotundus* against *Pheretima posthuma* was reported by Kasala et al. [22]. In addition, *C. rotundus* revealed potent activity against *E. histolytica* trophozoites in vitro and showed verified safety evidence for use [24].

In *Trichinella* spp., the cell wall includes the cuticle, hypodermis, and somatic musculature. Cuticle integrity is essential for parasite shape, protection, and nutrition and is necessary for osmoregulation [49].

In this study, electron microscopy scans demonstrated substantial adult worm destruction, loss of the normal morphology in groups treated with n-BuOH fr. and albendazole. It kept its typical appearance when incubated in the culture medium.

Transcuticular passive diffusion is the primary route by which drugs enter nematodes, followed by the worm’s surface being destroyed. Surface blebs indicated effective anti-parasitic activity because they were thought to be the worm’s replacement for its destroyed surface membrane [41, 61].

In the present research, we explored the therapeutic effect of n-BuOH fr. by administering n-BuOH fr., ABZ, and combined treatment (n-BuOH fr. and ABZ). All treated groups notably decreased the adult worm total count compared to the control-infected group. The best results were demonstrated by the group GVa, which received combined therapy and showed the best reduction of adult worms of *T. spiralis* with an efficacy of 91%, followed by GIIIa, which was administered albendazole with an efficacy of 88%, and GIVa that received n-BuOH fr. with a satisfactory percentage of reduction of 70%.

Concerning how drugs affect the muscle phase, a significant decrease in the mean larval count per gram muscle was reported in all treated groups. The best reduction was found in group GVb, which received combination therapy with an efficacy of 81%, followed by the mice group that received ABZ (GIIIb) with an efficacy of (76%), and the mice group that received n-BuOH fr. (GIVb), with a 65% reduction.

These findings were consistent with the results of Fahmy et al. [25], as they reported that the combined therapies of *C. rotundus* extract with the standard drugs (nitazoxanide and spiramycin) had the highest effectiveness against murine cryptosporidiosis and toxoplasmosis, respectively, followed by standard medications.

The albendazole effect on *T. spiralis* was reported in many previous studies, with a variance in efficacy [37, 47, 62, 63]. The variation in the efficacy of albendazole on intestinal and muscular phases was attributed to variance in treatment dose, time, and duration [63].

Albendazole acts mainly by inhibiting microtubule polymerization through selective binding to the parasite beta-tubulin monomer, besides having a small effect on host tubulin binding [64]. However, a study by Siriyasatien et al. [63] concluded that for the early stage of *T. spiralis* infection, 20 mg/kg albendazole given for 15 days was effective in treating infection in mice, while the late stage of infection was witnessed to be tolerant of albendazole. However, the duration of treatment was longer.

The result was similar to a study by McCracken [65], who documented that the *Trichinella* population became less susceptible to treatment when the worms matured. Comparing previous results with the results of the current study might explain the promising results found in the groups treated with the combined therapy.

In this study, the infected control group’s small intestinal sections underwent histological evaluation and revealed significant intravillous inflammatory cellular infiltration, primarily composed of lymphocytes and plasma cells. The intestinal villi showed broadening and atrophy. Besides, adult worm fragments were found in the intestinal lumen. The infected control group’s muscle tissue samples showed a massive amount of encysted *T. spiralis* larvae widely distributed in the sarcoplasm of muscle cells and several chronic inflammatory cells. These results agreed with that of El-Wakil et al. [41] and Dyab et al. [66]. There was an obvious reduction in damaging and inflammatory alterations in the treated groups. The group that received combination therapy had the most promising results in regaining typical architecture, having the fewest cysts with degenerating capsules, and focal pericapsular plasma-lymphocytic inflammatory cellular infiltration.

## Conclusion

The present study concluded that *C. rotundus* 90% MeOH extract, and its derived fractions had anti-trichinellosis activity. Their lethal effects were evident in in vitro studies on adult worms with the n-BuOH fr. proved to have the most promising effects. Moreover, the n-BuOH fr. demonstrated therapeutic effects, especially when co-administrated with albendazole. The anti-trichinellosis effect of n-BuOH and pet-ether fractions might be due to other secondary metabolites such as terpenoids, saponins, steroids, and alkaloids.

Additionally, 26 phenolic compounds were successfully characterized in 90% MeOH extract by the LC–ESI–MS technique in the negative ion mode. The identified compounds were classified into phenolic acids, flavonoids, and organic acids. The major compounds in this investigation are relative to apigenin and luteolin O or C-glycosides, which have a therapeutic effect. Therefore, these findings encourage us to further biological and chemical investigations of the active fractions, delineate their mechanisms of action, and support their use in pharmaceutical formulations.

## Supplementary Information

**Additional file 1.**

## Data Availability

The datasets generated or analyzed during the current study are available from the corresponding author upon reasonable request.

## Abbreviations

ABZ: Albendazole
C. rotundus: Cyprus rotundus
DMSO: Dimethyl sulfoxide
DW: Dry weight
EtOAc: Ethyl acetate
g: gram
GAE: Gallic acid equivalent
Kg: Kilogram
LC-ESI-MS: Liquid chromatography electrospray ionization mass spectrometry
MeOH: Methanol
mg: Milligram
n-BuOH: Normal butanol
pet-ether: Petroleum ether
RE: Rutin equivalent

## Declarations

The TBRI-REC approved the experimental research and field studies on plants, including the *Cyprus rotundus* collection, and they adhered to the relevant institutional, national, and international guidelines and legislation.
