# Supplementary material for: In vitro and in vivo anthelmintic and chemical studies of Cyperus rotundus L. extracts
Source: BMC Complement Med Ther. 2023 Jan 19;23:15. doi: 10.1186/s12906-023-03839-7 (PMC9850539; doi:10.1186/s12906-023-03839-7)
Supplement: Supplementary file 1 — Additional file 1. [file 12906_2023_3839_MOESM1_ESM.docx]

***In Vitro* and *In Vivo* Anthelmintic and Chemical Studies of *Cyperus rotundus L.* Extracts**

**Eman S. El-Wakil ^a*^, Shimaa Shaker^a^, Tarek Aboushousha^b^, El-Sayed S. Abdel-Hameed^c^, and Ezzat E.A. Osman^c^**

^a^ Department of Parasitology, Theodor Bilharz Research Institute, Kornaish El-Nile St., 12411, Giza, Egypt,

^b^ Department of Pathology, Theodor Bilharz Research Institute, Kornaish El-Nile St., 12411, Giza, Egypt,

^c^ Department of Medicinal Chemistry, Theodor Bilharz Research Institute, Kornaish El-Nile St., 12411, Giza, Egypt.

*** Corresponding author:

**Eman S. El-Wakil**

Department of Parasitology, Theodor Bilharz Research Institute, Kornaish El-Nile St., 12411, Giza, Egypt.

Tel: (+2) 01009928721

Fax: (+2) 0235408125

E-mail: drfaith@ymail.com

**LC-ESI-MS characterization of the main phytochemicals of *C. rotundus* 90% MeOH extract**

The electrospray ionization (ESI) and mass (MS) techniques combined with liquid chromatographic (LC) techniques have become a powerful approach in the structural assignment and quantification of the active ingredients in plant extracts [1]**.** The identified compounds included phenolic acids, flavonoids and organic acids.

Phenolic acids are one of the main classes of phenolic compounds found in *C. rotundus* extracts [2]**.** In this study, some of them were detected in 90 % MeOH extract including compound 1 (t_R_= 12.28 min) exhibited molecular ion peak at *𝑚/𝑧* 191 [M‑H]^‑^ and main product ion at *𝑚/𝑧* 173, which characteristic for quinic acid [3]. Compound 3 (t_R_= 16.96 min) showed the [M‑H]^‑^ ion at *m/z* 387 and yielded fragment ions at *m/z* 211 and 197, indicating that a glucuronide conjugate of the three methylation prod­ucts of gallic acid. Thus, it was tentatively characterized as trimethyl gallic acid-glucuronide [4]**.** Compounds 5, 7 and 8 (t_R_= 18.96, 23.10 and 23.77 min, respectively) possessed a molecular ion peak at *m/z* 353 [M-H]^-^ and their diagnostic product ions at *m/z* 191 and 179. As these three isomer compounds presented a base peak at *m/z* 191 (100%), the difference between them is based on the secondary peaks. The isomer 3-*O*- presented peaks at *m/z* 179 (63%) and 135 [caffeic acid- H-CO_2_]^-^ and the isomer 4-*O*- exhibited peaks at *m/z* 179 (13%) and 135. As well as the isomer 5-*O*- showed a characteristic product ion peak at *m/z* 161 [caffeic acid-H-H_2_O]^-^. Therefore, these compounds were tentatively identified as 3-*O*-, 4-*O*- and 5-*O*-cafeoylquinic acid (chlorogenic acid), respectively [5, 6].

Compounds 6 and 13 (t_R_= 21.63 and 37.12 min, respectively) afforded the molecular ion peak at *m*/*z* 593 [M-H]^−^ and other fragments at *m*/*z* 447 [M-H-146]^−^ correspond to loss of rhamnose unit, 285 [M-H- (146+162)]^−^ which means further loss of hexoside unit. Thus, these compounds were tentatively identified as luteolin-7-*O*-rutinoside and its isomer [7]. Compound 10 (t_R_= 32.71 min) exhibited the [M-H]^-^ ion at *m/z* 447, and produced other fragments at *m/z* 357 [M-90-H]^-^ and *m/z* 327[M-120-H]^-^ typical fragment ions of mono-C-glucoside. Thus, it was characterized as luteolin-8-C-glucoside or orientin [8]**.** Also, compound 15 (t_R_= 38.85 min) showed deprotonated molecule [M-H]^−^ at *m*/*z* 461 and base peak at *m*/*z* 285 [M-H-176]^−^, indicating the liberation of glucoronoid unit. Therefore, this compound was characterized as luteolin-7-*O-*glucoronoid. In addition, compound 22 (t_R_= 38.85 min) had a molecular ion peak at *m/z* 285 [M-H]^-^ and other fragments at m/z 199, 175 and 151, which is characteristic of luteolin [7, 9]**.**

Compound 9 (t_R_= 31.91 min) showed a molecular ion [M-H]^–^ at 𝑚/𝑧 563, which yielded main fragments at 𝑚/𝑧 473 [M-H-90]^–^, 𝑚/𝑧 443 [M-H-120]^–^. Meanwhile, accompanied with ions at 𝑚/𝑧 383 [M-H-120-60]^–^ and 𝑚/𝑧 353 [M-H-120-90]^–^. Therefore, it was unambiguously identified as apigenin-6-C-hexoside-8-C-pentoside. Moreover, these findings are matched with the literature data [10]. Compound 24 (t_R_= 54.48 min) had molecular ion peak at *m/z* 619 [M-H]^-^ and major fragments *m/z* 577 [M-H-42 (acetyl)]^-^, *m/z* 431 [M-H-42 (acetyl)- 146 (Rha)]^-^, *m/z* 269 [M-H-42 (acetyl)- 146 (Rha)- 162 (Glc)]^-^. Therefore, it was identified as apigenin-7-*O*-acetyl-rutinoside. Further, Compound 25 (t_R_= 56.35 min) affords a molecular ion peak at *m/z* 819 [M-H]^-^ and a base peak *m/z* 269 which corresponds to apigenin derivatives [11].

Compound 11 (t_R_= 33.78 min) had a molecular ion [M-H]^–^ at 𝑚/𝑧 563, which produced major fragments at 𝑚/𝑧 503 [M-H-60]^–^, 473 [M-H-90]^–^ and 𝑚/𝑧 443 [(M-H-120]^–^. Hence, it was established as isoschaftoside [10]. Compound 12 (t_R_= 36.58 min) gave the [M-H]^−^ ion at *m*/*z* 373 which yielded product ions at *m*/*z*  343 [M-H-2CH_3_]^−^, 328 [M-H-3CH_3_]^−^, and 300 [M-H-3CH_3_-CO]^−^. These data are characteristic for the known compound named as skullcapflavone II [12]. While, compounds 16, 19 and 23 (t_R_= 40.19, 42.06 and 51.40 min, respectively) afforded an [M-H]^−^ ion with *m/z* 373 and four fragments with *m/z* 358, 343, 328, and 313 means loss of four sequential methyl-groups. This fragmentation pattern is characteristic of quercetagetin-tetramethyl ester and its isomers [13]**.** Compounds 14 and 26 (t_R_= 37.65 and 59.42 min, respectively) gave a [M-H]^-^ molecular ion at *m/z* 343. The fragmentation pathway of this compound resulted in the loss of three methyl groups (-CH_3_) corresponding to product fragment ions [M-H- 343-328-313]^-^. Thus, these compounds were tentatively assigned as 5, 6-dihydroxy- 3',4',7-trimethoxyflavone and its isomer [14]**.**

Compound 17 (t_R_= 41.26 min) showed a deprotonated molecular ion [M-H]^-^ at *m/z* 637, with mass fragmentation pattern at *m/z* 491[M-H-146]^-^, indicating loss of rhamnose unit, *m/z* 329 [M-H-146- 162]^-^, indicating further loss of hexoside unit. This was followed by a major fragment ion at *m/z* 313. So, it was tentatively identified as tricin-*O*-rutionside. Compound 18 (t_R_= 41.66 min) showed [M-H]^-^ at *m/z* 653, with product ions at *m/z* 491[M-H-162]^-^ and *m/z* 329 [M-H-(2×162)]^-^, attributed to loss of two hexoside units. Thus, it was tentatively characterized as tricin-*O*-dihexosides. Moreover, compound 20 (t_R_= 43.26 min) had [M-H]^-^ at *m/z* 637, with product ions at *m/z* 505 [M-H-132]^-^, which means the elimination of pentoside unit and *m/z* 329 [M-H-132-176)]^-^, reflected loss of ferulyl unit. Thus, it was tentatively characterized as tricin-*O*-ferulyl-pentoside [15, 16]**.**

There was one organic acid detected in the 90% MeOH extract of *C. rotundus* which was tentatively identified as trihydroxy-octadecanoic acid with [M-H]- at *m/z* 329 with a typical fragmentation pattern at *m/z* 314 and 135 as described by Ben Said et al. [17].

**References**

1. Akther N, Andrabi K, Nissar A, Ganaie S, Chandan BK, Gupta AP, Khuswant M, Sultana S, Shaw AS. Hepatoprotective activity of LC-ESI-MS standardized Iris spuria rhizome extract on its main bioactive constituents. Phytomedicine. 2014; 21:1202–07.
2. Rana A, Negi PB, Sahoo NG. Phytochemical screening and characterization of bioactive compounds from Juniperus squamata root extract. Materials Today: Proceedings. 2022; 48: 672–675. <https://doi.org/10.1016/j.matpr.2021.07.305>.
3. Simirgiotis MJ, Benites J, Areche C, Sepúlveda B. Antioxidant Capacities and Analysis of Phenolic Compounds in Three *Endemic Nolana* Species by HPLC-PDA-ESI-M. Molecules. 2015; 20: 11490-11507; doi:10.3390/molecules200611490
4. Li F, Zhang Y‑B, Wei X, Song C‑H, Qiao M‑Q, Zhang H‑Y. Metabolic profiling of Shu‑Yu capsule in rat serum based on metabolic fingerprinting analysis using HPLC‑ESI‑MS^n^. Molecular Medicine Reports. 2016; 13: 419-4204.
5. Zhang Y, Shi P, Qu H., Cheng Y. Characterization of phenolic compounds in Erigeron breviscapus by liquid chromatography coupled to electrospray ionization mass spectrometry. Rapid Commun. Mass Spectrom. 2007; 21: 2971–2984. DOI: 10.1002/rcm.3166.
6. Faustino MV, Faustino MAF, Silva H, Cunha Â, Silva AMS, Pinto DCGA. *Puccinellia maritima, Spartina maritime,* and *Spartina patens* Halophytic Grasses: Characterization of Polyphenolic and Chlorophyll Profiles and Evaluation of Their Biological Activities. Molecules 2019; 24: 3796; doi:10.3390/molecules24203796.
7. Plazonić A, Bucar F, Maleš Ž, Mornar A, Nigović B, Kujundžić N. Identification and Quantification of Flavonoids and Phenolic Acids in Burr Parsley (*Caucalis platycarpos* L.), Using High-Performance Liquid Chromatography with Diode Array Detection and Electrospray Ionization Mass Spectrometry. Molecules. 2009; 14: 2466-2490. doi:10.3390/molecules14072466.
8. Hassan WHB, Abdelaziz S, Al Yousef HM. Chemical Composition and Biological Activities of the Aqueous Fraction of *Parkinsonea aculeata* L. Growing in Saudi Arabia. Arab.J.Chem. 2019; 12: 377–387.[https://bdoi.org/10.1016/j.arabjc.2018 08.003](https://bdoi.org/10.1016/j.arabjc.2018%2008.003).
9. Babiaka SB, Moumbock AFA, G¨unther S, Ntie-Kang F. Natural products in *Cyperus rotundus* L. (Cyperaceae): an update of the chemistry and pharmacological activities. RSC Adv. 2021; 11:15060.
10. Zhou C, Luo Y, Lei Z, Wei G. UHPLC-ESI-MS Analysis of Purified Flavonoids Fraction from Stem of *Dendrobium denneaum* Paxt. and Its Preliminary Study in Inducing Apoptosis of HepG2 Cells. Evid.-based Complement. Altern. Med. 2018; Article ID 8936307, 10 pages. <https://doi.org/10.1155/2018/8936307>.
11. Brito A, Ramirez JE, Areche C, Sepúlveda B, Simirgiotis MJ. HPLC-UV-MS Profiles of Phenolic Compounds and Antioxidant Activity of Fruits from Three Citrus Species Consumed in Northern Chile. Molecules. 2014; 19:17400-17421.doi:10.3390/ molecules191117400.
12. Zhang J, Yang M, Jiang B, Huang H, Chen G, Lu Z, Li X, Bi K, Guo D. Analysis of Major Chemical Constituents in Luan-Pao- Prescription Using Liquid Chromatography Coupled with Electrospray Ionization Mass Spectrometry. Nat. Prod. Commun. 2008; 3 (5): 697-704.
13. Olennikov DN, Chirikova NK, Kashchenko NI, Nikolaev VM, Kim S-W and Vennos C. Bioactive Phenolics of the Genus Artemisia (Asteraceae): HPLC-DAD-ESI-TQ-MS/MS Profile of the Siberian Species and Their Inhibitory Potential Against a-Amylase and a-Glucosidase. Front. Pharmacol. 2018; 9: 756. doi:10.3389/ fphar.2018.00756.
14. Salih EYA, Fyhrquist P, Abdalla AMA, Abdelgadir AY, Kanninen M, Sipi M, Luukkanen O, Fahmi MKM, Elamin MH, Ali HA. LC-MS/MS Tandem Mass Spectrometry for Analysis of Phenolic Compounds and Pentacyclic Triterpenes in Antifungal Extracts of Terminalia brownii (Fresen). Antibiotics (Basel). 2017; 6(4): 37. doi: 10.3390/antibiotics6040037.
15. Galland M, Boutet-Mercey S, Lounifi I, Godin B, Balzergue S, Grandjean O, Morin H, Perreau F, Debeaujon I, Rajjou L. Compartmentation and Dynamics of Flavone Metabolism in Dry and Germinated Rice Seeds. Plant Cell Physiol. 2014; 55(9): 1646–1659. doi:10.1093/pcp/pcu095
16. Kang J, Price W, Ashton J, Tapsell L, Johnson S. Identification and characterization of phenolic compounds in hydromethanolic extracts of *Sorghum wholegrains* by LC-ESI-MS^n^. Food Chemistry. 2016; 211: 215-226.
17. Ben Said R, Hamed AI, Mahalel UA, Al-Ayed AS, Kowalczyk M, Moldoch J, Oleszek W, Stochma A. Tentative Characterization of Polyphenolic Compounds in the Male Flowers of *Phoenix dactylifera* by Liquid Chromatography Coupled with Mass Spectrometry and DFT. Int. J. Mol. Sci. 2017; 18: 512; doi:10.3390 /ijms18030 512.
